# Design and stability analysis of the gear-type mobile mechanism with a single actuator

**DOI:** 10.1038/s41598-024-51248-8

**Published:** 2024-01-22

**Authors:** Kan Shi, Jianglong Tang, Jiachao Liu, Liang Yuan

**Affiliations:** 1 College of Mechanical and Electronic Engineering, Shandong University of Science and Technology, Qingdao, 266590 China; 2 College of Information Science and Technology, Beijing University of Chemical Technology, Beijing, 100089 China

**Keywords:** Engineering, Mechanical engineering

## Abstract

Gear mechanism transmits the motion and power of parallel axes, intersecting axes and staggered axes, which has been widely employed in production and life. Gear-type mobile mechanism is a type of robot that can achieve motion through gear transmission. Due to its unique motion mode, it can handle various tasks in certain unconventional environments, such as particularly steep surfaces. Cylindrical gear, bevel gear and non-cylindrical gear are taken as the main parts of the mechanism to form a novel research series, respectively. The models of gear-type mobile mechanism are established in this paper, and the degrees of freedom of the mechanism are briefly calculated based on the screw theory. Simultaneously, the influence of centroid trajectory on motion stability is discussed to solve the possible problem that opposite rotation occur during the movement. Furthermore, the trajectory model of zero moment point is calculated considering the motion of the gear-type mobile mechanism. After that, the simulation and experimental analysis of its motion capability show that the gear-type mobile mechanism has excellent characteristics in stability, flexibility and continuity.

## Introduction

Creatures on the Earth have evolved into various forms of motion, rolling is one of the most basic forms of motion in nature^1^. Some smaller animals move in a rolling manner to adapt to the environment^2,3^. Many mobile mechanisms have been designed after being inspired by these creatures^4–6^, in this paper, studying from many mature theories of the gear and mobile mechanism, and exploring innovative gear-type mobile mechanisms.

The innovation of mobile mechanisms can improve the motion ability of robots and expand their application range. A two degree of freedom (DOF) rolling mechanism based on the Bricard linkage mechanism^7^ and a novel underactuated tetrahedral mobile robot with 12 DOF^8^. The problems of impact and singular position of moving mechanism in the process of motion have always restricted its development. Liu has come up with two different mechanisms for these issues, respectively^9,10^. Wang et al. proposed a reconfigurable triangular prism mobile robot with multiple motion modes^11^. Hao proposed a mobile mechanism that relies on gravity and inertial forces. From a mechanism point of view, the overly complex structure of the connecting rod will lead to difficulty in control and poor stiffness^12^. However, the ingenious design of the mechanism as an integral closed-chain mechanism, such as an octagonal snowflake rolling mechanism, can significantly improve the stiffness and load-bearing capacity.All the parts of the spherical robot are enclosed in a spherical space. There is no side that cannot be recovered during the movement, and it can move in any direction with high flexibility^13–15^. In order to improve the mobility of the spherical robot on rough and flat roads, Chang et al. designed a mobile robot with jumping ability by combining the double hemispherical mobile mechanism and the five-bar linkage mechanism^16^. Mareket al. tried to equip spherical robots with different drive mechanisms and sensors to adapt to more applications^17^. Different from the above-mentioned rolling mobile mechanisms, Romanishin et al. proposed a mobile robot composed of magnetic cubes, which is driven by momentum and flips around the edges of its own cube when over obstacles^18^. Based on the spring loaded inverted pendulum dynamics model, Haoran et al. designed a monopod jumping robot^19^. Qu et al. proposed a novel type of mobile robot driven by magnetic force, which can play a role in the future medical field^20^.

Many scholars have conducted in-depth research on the stability of mobile robots^21^. In this respect, it can not only be realized by designing mechanical structures, but also be optimized from the stability algorithm level^22–24^.Wang et al. proposed four dynamic rolling motion schemes for a morphological rolling robot with a closed five-arc linkage mechanism and compared them^25^. Jing et al. studied the influence of the trajectory of the centroid on the stable walking of the mobile robot, and designed a control system that can adaptively adjust the centroid^26^. Alexander P made assumptions and discussions on the singularity of spherical robots, and the research results are helpful for the dynamic analysis of spherical robots^27^. At present, with the improvement of design theory and technical progress, the stability control of mobile robots is more and more accurate and reliable. But this also makes the complexity of mobile robots greatly increased.

With the increasing demand for transmission forms, research on gears is gradually deepening. Yang et al. used the multi degree of freedom conjugate surface meshing theory to discuss the conjugate tooth profile surface equation, the distribution law of gear teeth on the spherical surface, and the determination of gear tooth profile^28^. We were inspired by Pan et al.'s proposal for the application of ball crown gears in robot flexible wrists^29^. In this paper, the transmission mechanism is creatively applied as a mobile mechanism. A novel gear-type mobile mechanism is proposed, which further expands the application range of gears. The main advantages of the gear-type mobile mechanism are as follows: it only requires single power to drive, with simple control, high reliability and low energy consumption. In addition, the mobile mechanism always maintains surface contact with the ground when moving, and has higher static stability than the spherical mechanism.

## Mechanical design

### Configuration design

**Figure 1:**
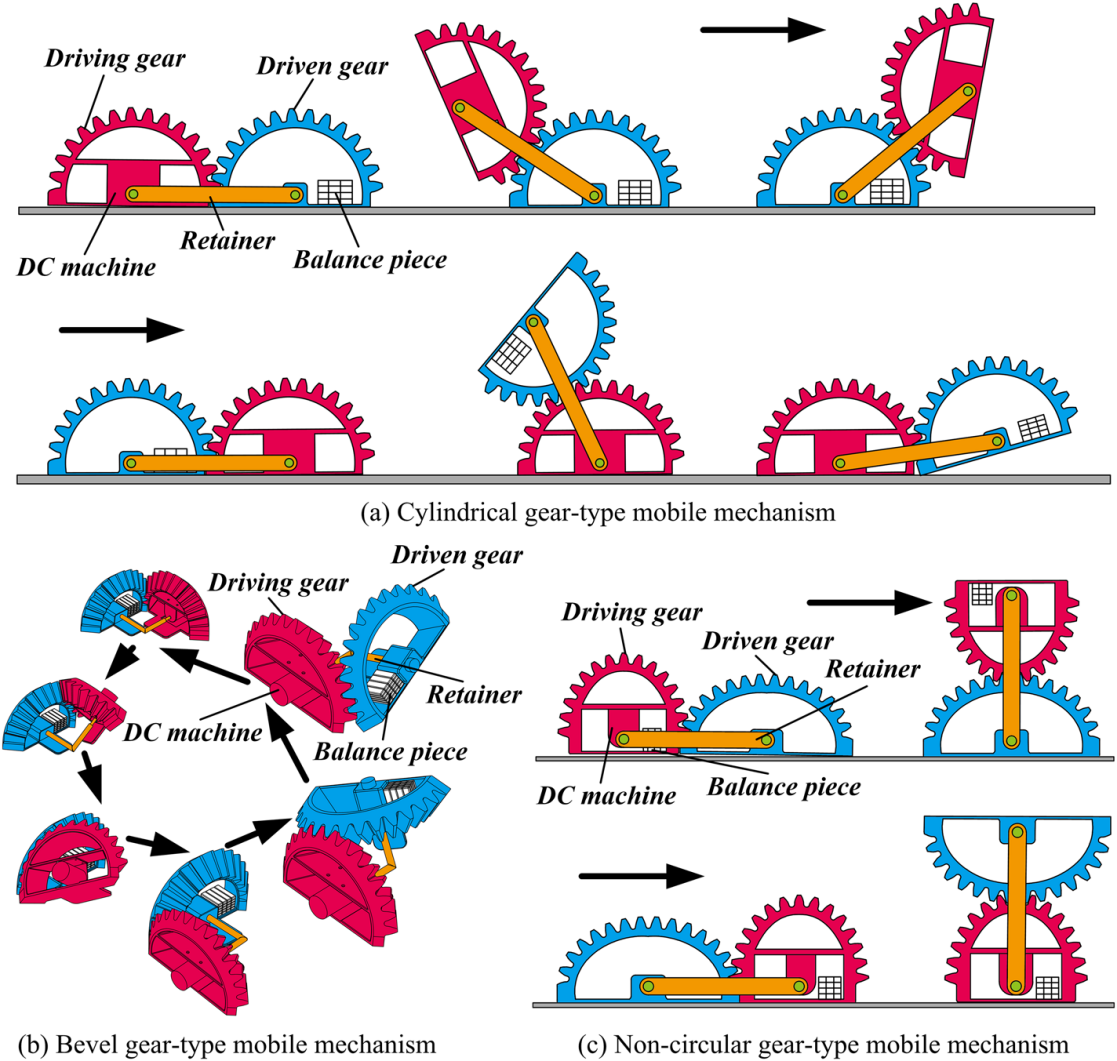
The gear-type mobile mechanism.

This paper proposes the concept of gear-type mobile mechanism, which subverts traditional way that gears can only be employed as transmission mechanisms. As an innovative concept of mobile mechanism, cylindrical gear-type mobile (CGM) mechanism can realize linear motion with a constant speed, bevel gear-type mobile (BGM) mechanism realizes steering motion, and non-circular gear-type mobile (NCGM) mechanism is able to realize variable speed linear motion. This is determined by the shape of pitch curve and tooth profile. The above gear-type mobile mechanism is mainly composed of five components, namely driving gear, driven gear, direct current (DC) machine, balance piece, and retainer, as shown in Fig. 1.

As shown in Fig. 1, the CGM mechanism consists of two semi-cylindrical gears. The two sides of the gear are the tooth groove surface and the gear tooth surface respectively, and the two semi-cylindrical gears are connected by retainers. The BGM mechanism consists of two semi-conical gears. Because the BGM mechanism tends to overturn toward the center during operation, a base plate is added to the bottom surface of the two bevel gears to increase the stability. The NCGM mechanism is composed of two semi-circular gears. Using the non-circular gear as the mobile mechanism, the main innovation is to directly realize the variable transmission ratio movement. The oval gear of order $n = 2$ has better continuity in the transmission system and the center of its rotating shaft is at the geometric centroid of the gear. This is of great significance to design mobile mechanism with symmetrical structure and smooth motion.

When the DC machine starts, the driven gear is used as the fixed platform first, and the driving gear is turned over from the driven gear until the lower plane of the two gears is parallel to the ground at the same time. Then the driving gear is used as the fixed platform, the driven gear is turned over from the driving gear. This is repeated to achieve continuous periodic motion of the gear-type mobile mechanism. The center distance between gears is determined by the retainer. Combined with the movement principle of parallel mobile mechanism, the gear-type mobile mechanism can realize the transformation of the moving platform and move directly at any transmission ratio. It is crucial to maintain reliable contact between ground and gear for achieving the rolling motion stably. Otherwise, the opposite rotation (OR) will occur when the gears are engaged. The OR between gears can be avoided by adjusting the structural parameters of the gear-type mobile mechanism. It is verified that they are able to realize uniform linear rolling, uniform circular rolling and variable linear rolling respectively.

### Degree of freedom analysis

The motion screw system and the inverse screw system of the gear-type mobile mechanism are established based on the screw theory. Using the modified Grübler Kutzbach (G-K) formula to analyze motion relationship between joints, and it is proved that the gear-type mobile mechanism proposed in this paper can be driven by only a single motor with one DOF.

**Figure 2:**
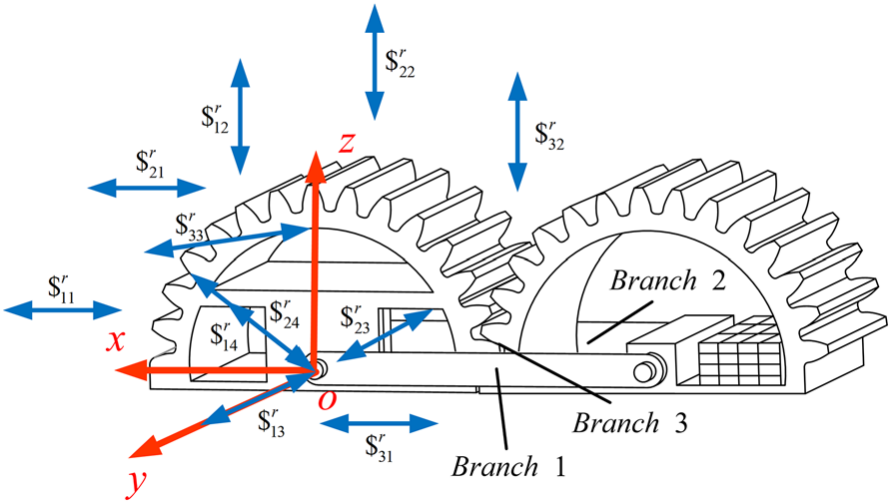
Analysis of cylindrical gear mobile mechanism DOF.

As shown in Fig. 2, taking the CGM mechanism as an example, it can be regarded as a parallel mechanism with three moving branch chains, i.e., *Branch* 1, *Branch* 2 and gears mesh with each other to form *Branch* 3. The Plucker coordinates of each moving branch chain are as follows:

1 $$\left\{ \begin{gathered} \$_{11} = (0, \, 1, \, 0; \, 0, \, 0, \, 0) \hfill \\ \$_{12} = (0, \, 1, \, 0; \, - c, \, 0, \, a) \hfill \\ \end{gathered} \right.$$

2 $$\left\{ \begin{gathered} \$_{{{21}}} { = (0, 1, 0; 0, 0, 0)} \hfill \\ \$_{22} { = (0, 1, 0; } - c{, 0, }a{)} \hfill \\ \end{gathered} \right.$$

3 $$\left\{ \begin{gathered} \$_{31} { = (0, 0, 0; 0, 1, 0)} \hfill \\ \$_{{{32}}} { = (0, 0, 0; }d{, 0, }e{)} \hfill \\ \$_{{{33}}} { = (0, 1, 0; } - e{, 0, }d{)} \hfill \\ \end{gathered} \right.$$

where $\$_{ij}$ represents the motion screw expression of the *j*-th moving pair in the *i*-th moving branch of the CGM mechanism. *Branch* 1 and *Branch* 2 provide two rotating pairs respectively, and the motion screw is shown in Eqs. (1–2). The gear engagement of *Branch* 3 can be equivalent to two kinematic pairs and one rotating pair, and the motion screw is shown in equation Eq. (3). Simultaneously, *a*, *c*, *d*, and *e* are related to the structural parameters and motion posture of gear-type mobile mechanism.

Then the inverse screws of each moving branch chain of the CGM mechanism are as follows:

4 $$\left\{ \begin{gathered} \$_{{{1}1}}^{r} = ({0, 0, 0; 1, 0, 0)} \hfill \\ \$_{{{1}2}}^{r} = ({0, 0, 0; 0, 0, 1)} \hfill \\ \$_{{{1}3}}^{r} = ({0, 1, 0; 0, 0, 0)} \hfill \\ \$_{{{1}4}}^{r} = (a{, 0, }c{; 0, 0, 0)} \hfill \\ \end{gathered} \right.$$

5 $$\left\{ \begin{gathered} \$_{{{2}1}}^{r} = ({0, 0, 0; 1, 0, 0)} \hfill \\ \$_{{{2}2}}^{r} = (0{, 0, 0; 0, 0, 1)} \hfill \\ \$_{{{2}3}}^{r} = ({0, 1, 0; 0, 0, 0)} \hfill \\ \$_{{{2}4}}^{r} = (a{, 0, }c{; 0, 0, 0)} \hfill \\ \end{gathered} \right.$$

6 $$\left\{ \begin{gathered} \$_{31}^{r} = ({0, 0, 0; 1, 0, 0)} \hfill \\ \$_{32}^{r} = (0{, 0, 0; 0, 0, 1)} \hfill \\ \$_{33}^{r} = (e{, - }d{, 0; 0, }e^{2} { + }d^{2} {, 0)} \hfill \\ \end{gathered} \right.$$

G–K formula:

7 $$M = (6 - \lambda )(n - g - 1) + \sum\limits_{i = 1}^{g} {f_{i} } + v$$

According to the analysis of the screw theory, the number of common constraints $\lambda$ is 2, the number of components $n$ is 4, the number of moving pairs $g$ is 5, the total number of DOF is 7, and the redundant constraints $v$ are 2, i.e.

8 $$M = (6 - 2) \times (4 - 5 - 1) + 7 + 2 = 1$$

The BGM mechanism has only two moving branch chains, which are perpendicular to each other. Therefore, the BGM mechanism has only one DOF to rotate around the *z-*axis, and the movements in the other directions are accompanying motions. The NCGM mechanism has only one DOF as well. Compared with the CGM mechanism, just the structural parameters of the gear are different, which does not affect the calculation result of the DOF. The innovation is that rolling with variable transmission ratio can be achieved by utilizing the transmission characteristics of non-circular gears themselves.

### Rolling locomotion analysis

#### Centroid coordinates of CGM mechanism and BGM mechanism

The centroid is defined as follows:

9 $$\left[ {\begin{array}{*{20}c} x & y & z \\ \end{array} } \right]^{{\varvec{T}}} = \frac{1}{{\sum\limits_{i = 1}^{n} {m_{i} } }}\left[ {\begin{array}{*{20}c} {\sum\limits_{i = 1}^{n} {\left( {m_{i} x_{i} } \right)} } & {\sum\limits_{i = 1}^{n} {\left( {m_{i} y_{i} } \right)} } & {\sum\limits_{i = 1}^{n} {\left( {m_{i} z_{i} } \right)} } \\ \end{array} } \right]^{{\varvec{T}}}$$

where $$\left[ {\begin{array}{*{20}c} x & y & z \\ \end{array} } \right]^{{\varvec{T}}}$$ represent the coordinates of the centroid,$x_{i} ,y_{i} ,z_{i}$ is the coordinates of each part, and $m_{i}$ is the mass of each part.

**Figure 3:**
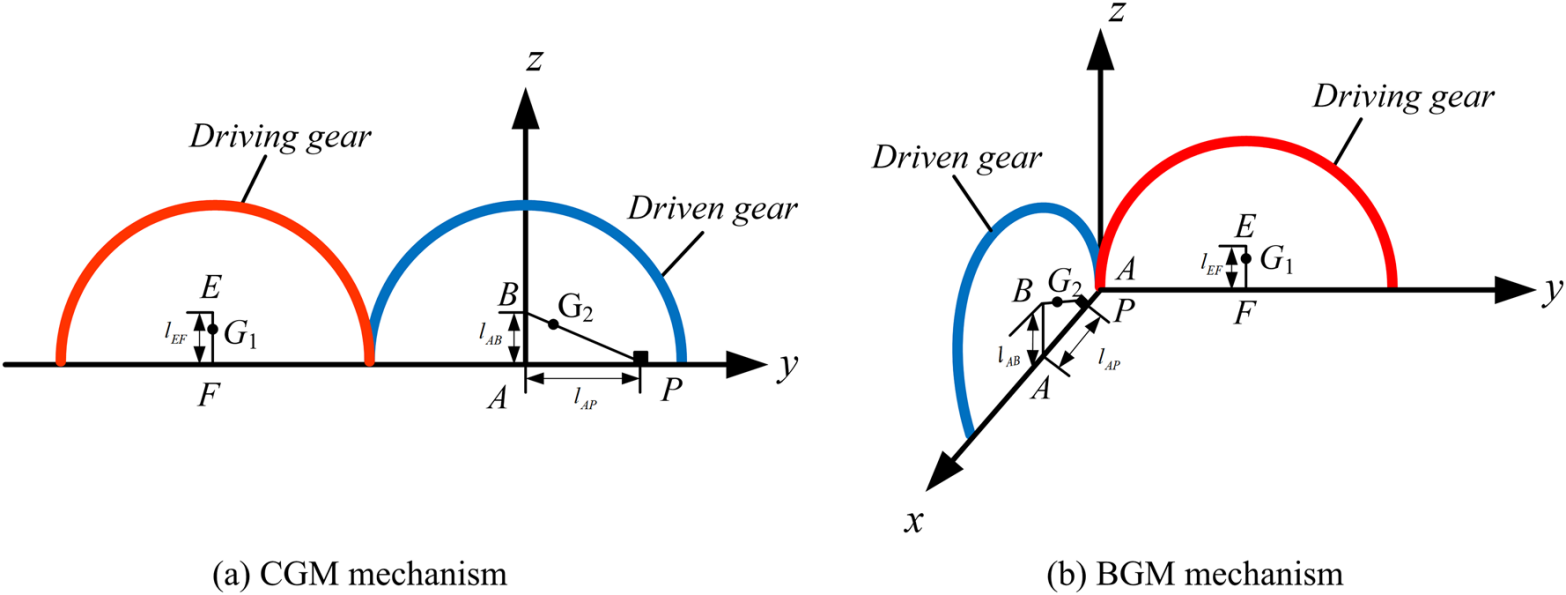
Centroid distribution of the gear-type mechanism.

Figure 3 shows a schematic diagram of the CGM mechanism and BGM mechanism. For the convenience of explanation, the driving gear is sign as gear 1 and the driven gear is sign as gear 2. *A* is the coordinate origin, the *y*-axis direction is along the tooth groove surface of gear 1, the vertical direction is the *z*-axis direction, and the *x*-axis direction conforms to the right-handed coordinate system. *E* represents the centroid of gear 1, *B* represents the centroid of gear 2, and *P* represents the centroid of balance piece, *F* represents the centroid of DC machine.

The centroid coordinate of gear mechanism in *z*-axis direction is as follows:

10 $$l_{EF} = l_{AB} = \frac{1}{m}\sum\limits_{i = 1}^{n} {\left( {m_{i} z_{i} } \right)} = \frac{1}{m}\int {zdm = \frac{1}{m}\int {r\sin \theta dm} } = \frac{4R}{{3\pi }}$$

where $m$ is the overall quality of the gear,$r$ and $\theta$ are the parameters in polar coordinates system;$R$ is the generalized numerical radius, which is the maximum pitch curve radius for CGM mechanism and BGM mechanism.

As shown in Fig. 3a, the centroid $${\varvec{G}}_{i} = \left[ {\begin{array}{*{20}c} {x_{i} } & {y_{i} } & {z_{i} } \\ \end{array} } \right]^{{\text{T}}}$$($i = {1,2}$) of the CGM mechanism can be expressed as follows:

11 $$\left\{ \begin{gathered} \user2{ G}_{1} = \left[ {\begin{array}{*{20}c} {x_{1} } \\ {y_{1} } \\ {z_{1} } \\ \end{array} } \right] = \frac{1}{{m_{E} + m_{F} }}\left[ {\begin{array}{*{20}c} 0 \\ {y_{E} m_{E} + y_{F} m_{F} } \\ {z_{E} m_{E} + z_{F} m_{F} } \\ \end{array} } \right] \hfill \\ \user2{ G}_{2} = \left[ {\begin{array}{*{20}c} {x_{2} } \\ {y_{2} } \\ {z_{2} } \\ \end{array} } \right] = \frac{1}{{m_{B} + m_{P} }}\left[ {\begin{array}{*{20}c} 0 \\ {y_{B} m_{B} + y_{P} m_{P} } \\ {z_{B} m_{B} + z_{P} m_{P} } \\ \end{array} } \right] \hfill \\ \end{gathered} \right.$$

where,$m_{E}$ and $m_{B}$ represent the mass of gear 1 and gear 2, respectively; $m_{F}$ and $m_{P}$ represent the mass of DC machine and balance piece, respectively; $y_{E}$, $y_{F}$, $y_{B}$, $y_{P}$ represent horizontal ordinates of *E*, *F*, *B* and *P*;$z_{E}$, $z_{F}$, $z_{B}$, $z_{P}$ represents vertical coordinates of *E*, *F*, *B*, and *P*.

It is found in the experiment that the BGM mechanism is easier to dump the small end of bevel gear during the movement. Therefore, the BGM mechanism is simplified as the small end in the analysis. The centroid of BGM mechanism can be expressed as follows:

12 $$\left\{ \begin{gathered} \user2{ G}_{1} = \frac{{1}}{{m_{E} + m_{F} }}\left[ {\begin{array}{*{20}c} {x_{E} m_{E} + x_{F} m_{F} } \\ {y_{E} m_{E} + y_{F} m_{F} } \\ {z_{E} m_{E} + z_{F} m_{F} } \\ \end{array} } \right] \hfill \\ \user2{ G}_{2} = \frac{{1}}{{m_{B} + m_{P} }}\left[ {\begin{array}{*{20}c} {x_{B} m_{B} + x_{P} m_{P} } \\ {y_{B} m_{B} + y_{P} m_{P} } \\ {z_{B} m_{B} + z_{P} m_{P} } \\ \end{array} } \right] \hfill \\ \end{gathered} \right.$$

The symbolic representation of CGM mechanism also applies to BGM mechanism.

The complete motion process of CGM mechanism includes two sub cycles, namely, the gear 1 rolls around the gear 2 and the gear 2 rolls around the gear 1. The DC motor is running at an angular velocity of $\omega$, the length of the retainer is $2R$, and the angle has turned $\theta$($0^\circ \le \theta \le 180^\circ$) in time $t$.

When the gear 1 rotates around the gear 2, the gear 1 is a fixed platform, which keeps contact with the horizontal surface, and the gear 2 acts as a moving platform. The coordinates of *E*, *F*, *B* and* P* on the CGM mechanism are as follows:

13 $$\left\{ \begin{gathered} \user2{ r}_{E} = \left[ {\begin{array}{*{20}c} {x_{E} } & {y_{E} } & {z_{E} } \\ \end{array} } \right]^{{\varvec{T}}} = \left[ {\begin{array}{*{20}c} {0} & { - 2R{\varvec{cos}}\theta + l_{EF} {\varvec{sin}}2\theta } & {2R{\varvec{sin}}\theta + l_{EF} {\varvec{cos}}2\theta } \\ \end{array} } \right]^{{\varvec{T}}} \hfill \\ \user2{ r}_{F} = \left[ {\begin{array}{*{20}c} {x_{F} } & {y_{F} } & {z_{F} } \\ \end{array} } \right]^{{\varvec{T}}} = \left[ {\begin{array}{*{20}c} {0} & { - {2}R{\varvec{cos}}\theta } & {{2}R{\varvec{sin}}\theta } \\ \end{array} } \right]^{{\varvec{T}}} \hfill \\ \user2{ r}_{B} = \left[ {\begin{array}{*{20}c} {x_{B} } & {y_{B} } & {z_{B} } \\ \end{array} } \right]^{{\varvec{T}}} = \left[ {\begin{array}{*{20}c} {0} & 0 & {l_{AB} } \\ \end{array} } \right]^{{\varvec{T}}} \hfill \\ \user2{ r}_{P} = \left[ {\begin{array}{*{20}c} {x_{P} } & {y_{P} } & {z_{P} } \\ \end{array} } \right]^{{\varvec{T}}} = \left[ {\begin{array}{*{20}c} {0} & {l_{AP} } & 0 \\ \end{array} } \right]^{{\varvec{T}}} \hfill \\ \end{gathered} \right.$$

where $l_{EF}$,$l_{AB}$ and $l_{AP}$ represent the distance between their two points, as shown in Fig. 3.

The tooth groove surface of gear 1 contacts the horizontal surface again when *F* rotates $180^\circ$ with* A* as the center and 2*R* as the radius. Then the next cycle starts, gear 1 is stationary as a fixed platform, and gear 2 turns over along its tooth surface. In this process, the centroid coordinates of gear-type mobile mechanism can be expressed as follows:

14 $$\left\{ \begin{gathered} \user2{ r}_{B} = \left[ {\begin{array}{*{20}c} {x_{B} } & {y_{B} } & {z_{B} } \\ \end{array} } \right]^{{\varvec{T}}} = \left[ {\begin{array}{*{20}c} {0} & { - 2R{\varvec{cos}}\theta + l_{AB} {\varvec{sin}}2\theta + 2R} & {2R{\varvec{sin}}\theta + l_{AB} {\varvec{cos}}2\theta } \\ \end{array} } \right]^{{\varvec{T}}} \hfill \\ \user2{ r}_{P} = \left[ {\begin{array}{*{20}c} {x_{P} } & {y_{P} } & {z_{P} } \\ \end{array} } \right]^{{\varvec{T}}} = \left[ {\begin{array}{*{20}c} {0} & { - 2R{\varvec{cos}}\theta + l_{AP} {\varvec{cos}}2\theta + 2R} & {2R{\varvec{sin}}\theta - l_{AP} {\varvec{sin}}2\theta } \\ \end{array} } \right]^{{\varvec{T}}} \hfill \\ \user2{ r}_{E} = \left[ {\begin{array}{*{20}c} {x_{E} } & {y_{E} } & {z_{E} } \\ \end{array} } \right]^{{\varvec{T}}} = \left[ {\begin{array}{*{20}c} {0} & {2R} & {l_{EF} } \\ \end{array} } \right]^{{\varvec{T}}} \hfill \\ \user2{ r}_{F} = \left[ {\begin{array}{*{20}c} {x_{F} } & {y_{F} } & {z_{F} } \\ \end{array} } \right]^{{\varvec{T}}} = \left[ {\begin{array}{*{20}c} {0} & {2R} & 0 \\ \end{array} } \right]^{{\varvec{T}}} \hfill \\ \end{gathered} \right.$$

In Eq. (9), the whole centroid of the CGM mechanism is as follows:

15 $${\varvec{G}} = \left[ {\begin{array}{*{20}c} X & Y & Z \\ \end{array} } \right]^{{\varvec{T}}} = \frac{1}{{m_{1} + m_{2} + m_{3} }}\left[ {\begin{array}{*{20}c} {0} & {m_{1} y_{1} + m_{2} y_{2} - m_{3} R\cos \theta } & {m_{1} z_{1} + m_{2} z_{2} + m_{3} R\sin \theta } \\ \end{array} } \right]^{{\varvec{T}}}$$

where $m_{1}$ is the mass of the gear 1, including $m_{E}$, $m_{F}$; $m_{2}$ is the mass of the gear 2, including $m_{B}$ and $m_{P}$; $m_{3}$ is the mass of retainer.

It is found in the experiment that the BGM mechanism is easier to dump the small end of bevel gear during the movement. Therefore, the BGM mechanism is simplified as the small end in the analysis. As shown in Fig. 3b, the centroid $${\varvec{G}}_{i} = \left[ {\begin{array}{*{20}c} x & y & z \\ \end{array} } \right]^{{\text{T}}} \left( {i = {1,2}} \right)$$ of the BGM mechanism can be expressed as follows:

During the moving process of BGM mechanism, the trajectory of centroid coordinate will be an irregular space curve. When the gear 1 is fixed and the gear 2 rotates around it, the trajectory coordinates of *B*, *P*, *E* and *F* can be established in the space coordinate system as follows:

16 $$\left\{ \begin{gathered} \user2{ r}_{B} = \left[ {\begin{array}{*{20}c} {R - l_{AB} {\text{sin}}\theta } & {R - R{\text{cos}}\theta + l_{AB} {\text{cos}}\theta {\text{sin}}\theta } & {R{\text{sin}}\theta + l_{AB} {\text{cos}}\theta {\text{cos}}\theta } \\ \end{array} } \right]^{{\varvec{T}}} \hfill \\ \user2{ r}_{P} = \left[ {\begin{array}{*{20}c} {R - l_{AP} {\text{cos}}\theta } & {R - R{\text{cos}}\theta + l_{AP} {\text{sin}}\theta {\text{sin}}\theta } & {R{\text{sin}}\theta - l_{AP} {\text{sin}}\theta {\text{cos}}\theta } \\ \end{array} } \right]^{{\varvec{T}}} \hfill \\ \user2{ r}_{E} = \left[ {\begin{array}{*{20}c} {0} & R & {l_{EF} } \\ \end{array} } \right]^{{\varvec{T}}} \hfill \\ \user2{ r}_{F} = \left[ {\begin{array}{*{20}c} {0} & R & 0 \\ \end{array} } \right]^{{\varvec{T}}} \hfill \\ \end{gathered} \right.$$

The coordinates of *E*, *F*, *B*, *P*in the next half cycle can be expressed as follows:

17 $$\left\{ \begin{gathered} \user2{ r}_{E} = \left[ {\begin{array}{*{20}c} {R - R{\text{cos}}\theta + l_{EF} {\text{cos}}\theta {\text{sin}}\theta } & {R + l_{EF} {\text{sin}}\theta } & {R{\text{sin}}\theta + l_{EF} {\text{cos}}\theta {\text{cos}}\theta } \\ \end{array} } \right]^{{\varvec{T}}} \hfill \\ \user2{ r}_{F} = \left[ {\begin{array}{*{20}c} {R - R{\text{cos}}\theta } & R & {R{\text{sin}}\theta } \\ \end{array} } \right]^{{\varvec{T}}} \hfill \\ \user2{ r}_{B} = \left[ {\begin{array}{*{20}c} R & {2R} & {l_{AB} } \\ \end{array} } \right]^{{\varvec{T}}} \hfill \\ \user2{ r}_{P} = \left[ {\begin{array}{*{20}c} {R + l_{AP} } & {2R} & 0 \\ \end{array} } \right]^{{\varvec{T}}} \hfill \\ \end{gathered} \right.$$

Compared with the CGM mechanism, the BGM mechanism only needs one retainer. And half of retainer structure is always in the fixed state of the centroid during movement period. The mass of the remaining moving parts will be small, so it can be assumed that the mass of this parts are ignored. As a result, the centroid of BGM mechanism is as follows:

18 $${\varvec{G}} = \left[ {\begin{array}{*{20}c} X & Y & Z \\ \end{array} } \right]^{{\varvec{T}}} = \frac{1}{{m_{1} + m_{2} }}\left[ {\begin{array}{*{20}c} {m_{2} x_{2} + m_{1} x_{1} } & {m_{2} y_{2} + m_{1} y_{1} } & {m_{1} z_{1} + m_{2} z_{2} } \\ \end{array} } \right]^{{\varvec{T}}}$$

#### Analysis of NCGM mechanism

The transmission ratio of the non-circular gear is related to the shape of pitch curve, and it is not a constant for the gear train during the movement. The transmission ratio function of the non-circular gear designed in this paper is as follows:

19 $$i_{12} = \frac{{{\varvec{\omega}}_{{1}} }}{{{\varvec{\omega}}_{{2}} }} = \frac{{1 + k^{2} - 2k\cos 2\theta_{1} }}{{1 - k^{2} }}$$

where $k$ is the eccentricity of gear pitch curve, and $\theta_{1}$ is the angle that the gear 1 rotates in time $t$.${\varvec{\omega}}_{{1}}$ and ${\varvec{\omega}}_{{2}}$ represent the angular speeds of the gear 1 and gear 2 respectively.

**Figure 4:**
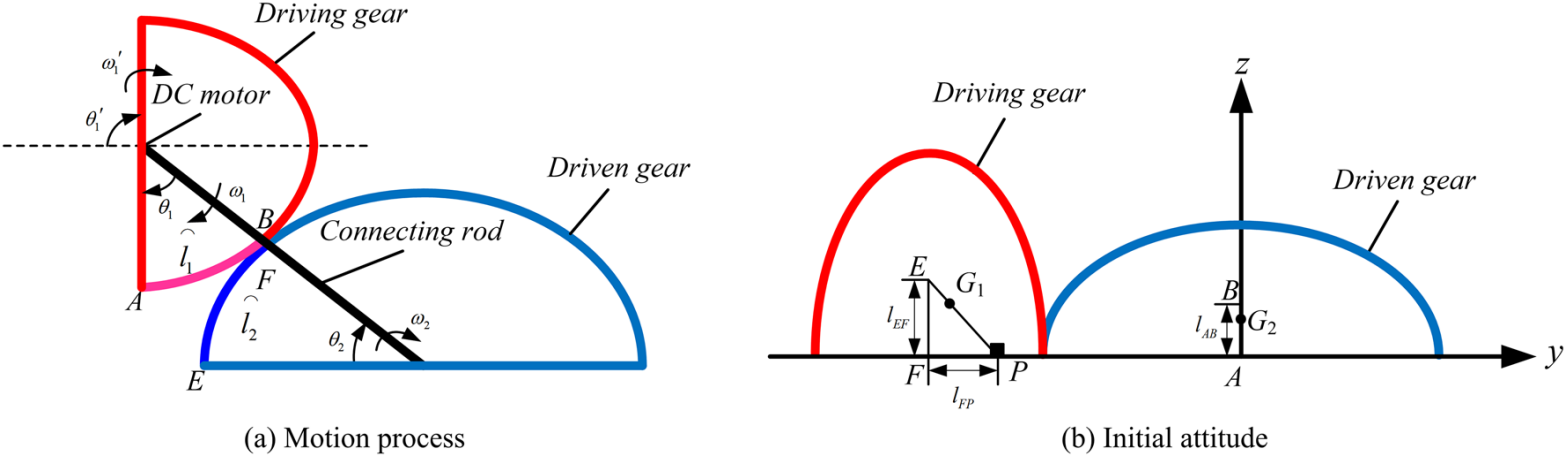
Motion analysis and initial attitude of the NCGM mechanism.

The movement of non-circular gear as a mobile mechanism is shown in Fig. 4a. This is similar to the fact that planet rotates itself while moving around the star. The gear 1 rotates around its own rotation axis with an angular velocity of $${\varvec{\omega}}_{{1}}$$, and the rotation angle within the time $$t$$ is $$\theta_{{1}}$$. The gear 1 revolves around the gear 2 at an angular velocity of $${\varvec{\omega}}_{{2}}$$, and the rotation angle within the time $$t$$ is $$\theta_{{2}}$$. Pure rolling motion is carried out between the pitch curves of two semi-gears, therefore, the rolling arc lengths are equal, i.e. $$\overset{\lower0.5em\hbox{$\smash{\scriptscriptstyle\frown}$}}{l}_{1} = \overset{\lower0.5em\hbox{$\smash{\scriptscriptstyle\frown}$}}{l}_{2}$$.

Taking the gear 1 as moving platform and gear 2 as fixed platform, the angular velocity ${\varvec{\omega}}_{1}^{\prime }$ of moving platform can be obtained as follows:

20 $${\varvec{\omega}}_{1}^{\prime } = {\varvec{\omega}}_{1} + {\varvec{\omega}}_{2} = (1 + i_{21} ){\varvec{\omega}}_{1} = (\frac{{1 - k^{2} }}{{1 + k^{2} - 2k\cos 2\theta_{{1}} }} + 1){\varvec{\omega}}_{1}$$

The angular displacement $\theta_{1}^{\prime }$ of moving platform is as follows:

21 $$\theta_{1}^{\prime } = \theta_{1} + \theta_{2} = \int_{0}^{{\theta_{1} }} {(\frac{{1 - k^{2} }}{{1 + k^{2} - 2k\cos 2\theta }} + 1)} d\theta$$

As shown in Fig. 4b, the centroid $${\varvec{G}}_{i} = \left[ {\begin{array}{*{20}c} x & y & z \\ \end{array} } \right]^{{\text{T}}} \left( {i = {1,2}} \right)$$ of the NCGM mechanism can be expressed as follows:

22 $$\left\{ \begin{gathered} \user2{ G}_{1} = \frac{1}{{m_{P} + m_{E} + m_{F} }}\left[ {\begin{array}{*{20}c} 0 \\ {y_{P} m_{P} + y_{E} m_{E} + y_{F} m_{F} } \\ {z_{P} m_{P} + z_{E} m_{E} + z_{F} m_{F} } \\ \end{array} } \right] \hfill \\ \user2{ G}_{2} = \left[ {\begin{array}{*{20}c} 0 \\ {y_{B} } \\ {z_{B} } \\ \end{array} } \right] \hfill \\ \end{gathered} \right.$$

The coordinates of *P*, *E*, and *F* can be determined as follows:

23 $$\left\{ \begin{gathered} \user2{ r}_{P} = \left[ {\begin{array}{*{20}c} {0} & { - {(}a + b{\text{)cos}}\theta_{2} + l_{FP} \cos \theta_{1}^{\prime } } & {{(}a + b{\text{)sin}}\theta_{2} - l_{FP} \sin \theta_{1}^{\prime } } \\ \end{array} } \right]^{{\varvec{T}}} \hfill \\ \user2{ r}_{E} = \left[ {\begin{array}{*{20}c} {0} & { - {(}a + b{\text{)cos}}\theta_{2} + l_{EF} \sin \theta_{1}^{\prime } } & {{(}a + b{\text{)sin}}\theta_{2} + l_{EF} \cos \theta_{1}^{\prime } } \\ \end{array} } \right]^{{\varvec{T}}} \hfill \\ \user2{ r}_{F} = \left[ {\begin{array}{*{20}c} {0} & { - {(}a + b{\text{)cos}}\theta_{2} } & {{(}a + b{\text{)sin}}\theta_{2} } \\ \end{array} } \right]^{{\varvec{T}}} \hfill \\ \user2{ r}_{B} = \left[ {\begin{array}{*{20}c} {0} & 0 & {l_{AB} } \\ \end{array} } \right]^{{\varvec{T}}} \hfill \\ \end{gathered} \right.$$

where *a* is the radius of the major axis of the pitch curve of the non-circular gear, and *b* is the radius of the minor axis.

When gear 2 is applied as the moving platform, the trajectory of centroid is as follows:

24 $$\left\{ \begin{gathered} \user2{ r}_{B} = \left[ {\begin{array}{*{20}c} {0} & {{(}a + b{)(}1 - {\text{cos}}\theta_{{1}} {)} + l_{AB} {\text{sin}}\theta_{2}^{\prime } } & {{(}a + b{\text{)sin}}\theta_{{1}} + l_{AB} {\text{cos}}\theta_{2}^{\prime } } \\ \end{array} } \right]^{{\varvec{T}}} \hfill \\ \user2{ r}_{P} = \left[ {\begin{array}{*{20}c} {0} & {a + b + l_{FP} } & 0 \\ \end{array} } \right]^{{\varvec{T}}} \hfill \\ \user2{ r}_{E} = \left[ {\begin{array}{*{20}c} {0} & {a + b} & {l_{EF} } \\ \end{array} } \right]^{{\varvec{T}}} \hfill \\ \user2{ r}_{F} = \left[ {\begin{array}{*{20}c} {0} & {a + b} & 0 \\ \end{array} } \right]^{{\varvec{T}}} \hfill \\ \end{gathered} \right.$$

The whole centroid of the NCGM mechanism is as follows:

25 $${\varvec{G}} = \left[ {\begin{array}{*{20}c} X & Y & Z \\ \end{array} } \right]^{{\varvec{T}}} = \frac{1}{{m_{1} + m_{2} }}\left[ {\begin{array}{*{20}c} 0 & {m_{2} y_{2} + m_{1} y_{1} } & {m_{1} z_{1} + m_{2} z_{2} } \\ \end{array} } \right]^{{\varvec{T}}}$$

where $m_{1}$ is the mass of gear 1, including $m_{P}$,$m_{E}$ and $m_{F}$.$m_{2}$ is the mass of gear 2, including $m_{B}$.

#### Analysis of the whole centroid of gear-type mobile mechanism

**Figure 5:**
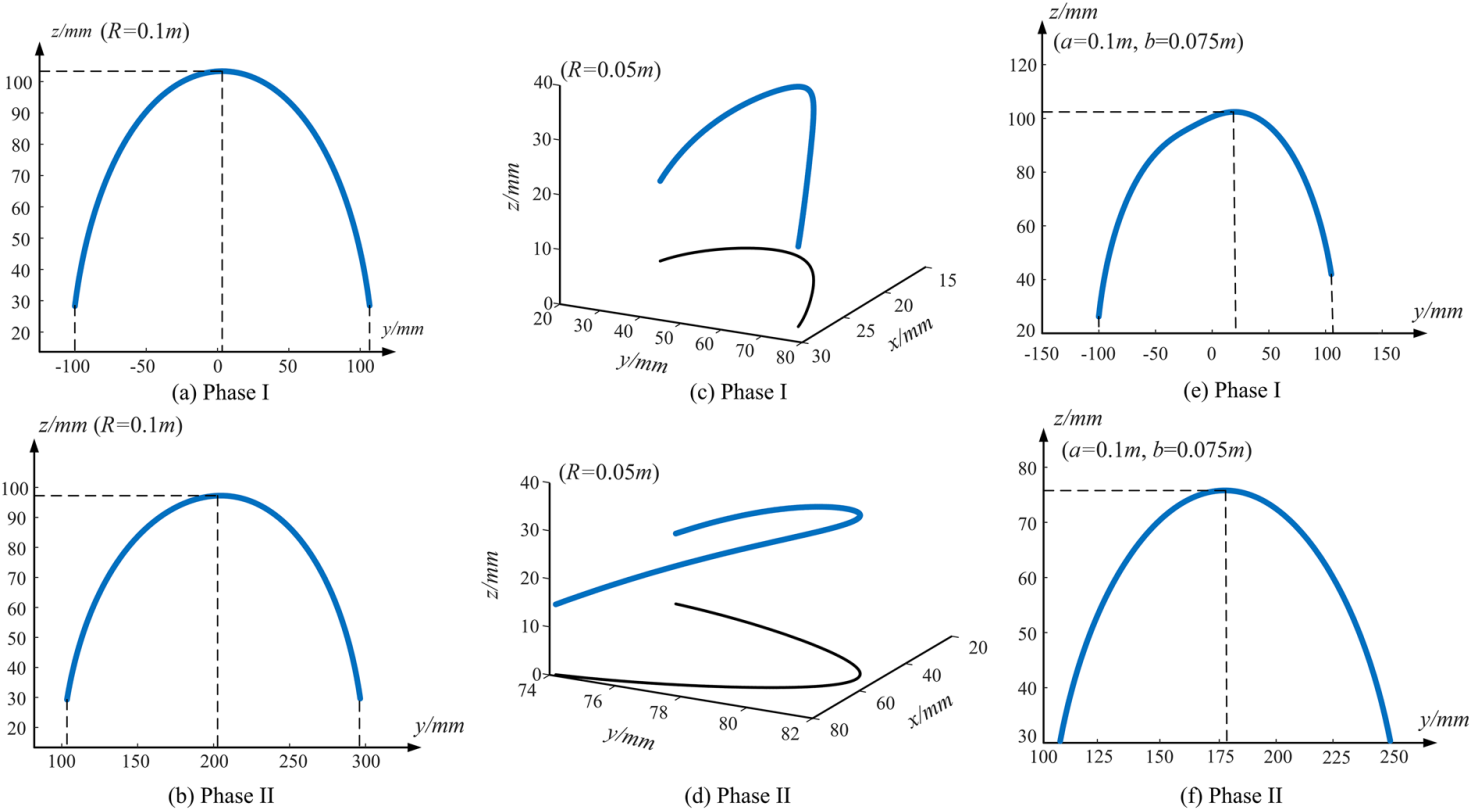
Centroid trajectory of the gear-type mobile mechanism.

Figure 5 shows the whole centroid trajectory of gear-type mobile mechanism. Take the mass of $m_{P}$ an $m_{F}$ as 0.2*kg*and 0.25* kg* respectively, $m_{E} \user2{ = }m_{B} \user2{ = 0}\user2{.5}kg$, $m_{3} = 0.05kg$, $l_{AP} = 0.025m$. Figure 5 a,c,e are the Phase I of gear-type mobile mechanism, indicating that gear 1 rotates around gear 2. Figure 5b,d,f are the Phase II of gear-type mobile mechanism, indicating that gear 2 rotates around gear 1.

For CGM, the support area of the first half cycle is $\left( { - 100\;{\text{mm}},100\;{\text{mm}}} \right)$ and the support area of the second half cycle is $\left( {100\;{\text{mm}},300\;{\text{mm}}} \right)$. DC motor can be started smoothly when $y \ge - 100\;{\text{mm}}$ at the beginning. Otherwise, the gear-type mobile mechanism cannot work normally, resulting in opposite rotation. Simultaneously, the impact effect will occur when the movement process is not completed in the extreme position of the support area. And the greater the value of $z$, the more obvious the impact effect. As shown in Fig. 5a,b, similarly the CGM mechanism designed according to above parameters can not only ensure smooth starting at the initial stage, but also avoid impact at the final stage.

As shown in Fig. 5c,d, the trajectory centroid coordinate of BGM mechanism in the $xAy$ plane is $\left\{ {(x,y)|16 \le x \le 27,25 \le y \le 74} \right\}$ and $\left\{ {(x,y)|27 \le x \le 79,74 \le y \le 81} \right\}$.Obviously, at first half cycle, the projection coordinate in *y*-axis direction is always within the support area $\left( { - 100\;{\text{mm}},100\;{\text{mm}}} \right)$. Simultaneously, it can be seen that the centroid of mechanism has moved to the small end of bevel gear, i.e. direction of *y*-axis, which makes the BGM mechanism dump to the side of centroid. Therefore, the design of BGM mechanism not only needs to consider the problem of opposite rotation between gears, but also needs to set base plate at the small end side of bevel gear. As a result, the setting of balance plate is indispensable, and the centroid of BGM mechanism is always located in support area. The motion stability is better than CGM mechanism, it starts stably and has no impact when landing.

It can be seen from Fig. 5e,f that the non-circular gear mobile mechanism can also be started and landed normally. However, it should be noted that under the design parameters of the mechanism, the motion of NCGM in the first cycle has a centroid fluctuation. This means that the smoothness of motion will be reduced, and compared with cylindrical gear type mobile mechanism, its impact on the ground is more obvious.

### Dynamic stability analysis of the geared mobile mechanism

#### Dynamic stability

The mechanism has no speed and acceleration when the mechanism is in static state. If the projection of centroid is on the support surface, it can generally be considered that mobile mechanism is in a stable state at this moment. However, the gear-type mobile mechanism will be affected by inertial force in the real environment, and the centroid of mechanism will change during the operation. Therefore, dynamic stability analysis is required in this paper.

Assuming that the mass of each part of mobile mechanism is $m_{i}$, and the centroid coordinate is $(x_{i} , \, y_{i} , \, z_{i} )$. The resultant force ***F*** of gravity and inertial force is as follows:

26 $${\varvec{F}} = \left[ \begin{gathered} F_{x} \hfill \\ F_{y} \hfill \\ F_{z} \hfill \\ \end{gathered} \right] = - \sum\limits_{i = 1}^{n} {m_{i} \left[ \begin{gathered} \ddot{x}_{i} \\ \ddot{y}_{i} \\ (\ddot{z}_{i} + g) \\ \end{gathered} \right]}$$

where $g$ is the acceleration of gravity in the direction parallel to *z*-axis. $\ddot{x}_{i}$, $\ddot{y}_{i}$ and $\ddot{z}_{i}$ are the second derivative of position coordinates of centroid respectively, i.e. component of acceleration on each coordinate axis.

The moment of the resultant force ***F*** on each coordinate axis is as follows:

27 $${\varvec{M}} = \left[ \begin{gathered} M_{x} \hfill \\ M_{y} \hfill \\ M_{z} \hfill \\ \end{gathered} \right] = - \sum\limits_{i = 1}^{n} {m_{i} } \left[ \begin{gathered} (\ddot{z}_{i} + g)y_{i} - \ddot{y}_{i} z_{i} \\ \ddot{x}_{i} z_{i} - (\ddot{z}_{i} + g)x_{i} \\ \ddot{y}_{i} x_{i} - \ddot{x}_{i} y_{i} \\ \end{gathered} \right]$$

Transfer the resultant force of reference coordinate system to the zero moment point (ZMP), and the resultant force components on *x-*axes and *y-*axes of ZMP are zero, i.e.

28 $$\left\{ \begin{gathered} M_{x} - F_{z} y_{zmp} = 0 \hfill \\ M_{y} - F_{z} x_{zmp} = 0 \hfill \\ \end{gathered} \right.$$

The CGM mechanism rolls in *yoz* plane,$y_{zmp}$ can be obtained in Eqs. (26–28) as follows:

29 $$y_{zmp} = \frac{{\sum\limits_{i = 0}^{n} {m_{i} (\ddot{z}_{i} + g)y_{i} - \sum\limits_{i = 0}^{n} {m_{i} \ddot{y}_{i} z_{i} } } }}{{\sum\limits_{i = 0}^{n} {m_{i} (\ddot{z}_{i} + g)} }}$$

The overall centroid and trajectory of the gear-type mobile mechanism have been obtained through static stability analysis, therefore,$y_{zmp}$ can be expressed as follows:

30 $$y_{zmp} = Y - \frac{{\ddot{Y} \cdot Z}}{{(\ddot{Z} + g)}}$$

where $\ddot{Z}$ is the acceleration of the centroid of entire mechanism in $Z$ direction and $\ddot{Y}$ is the acceleration in *y*-axes direction.

In Eqs. (11–15), the centroid coordinate of CGM has following relationship with the angular displacement of DC motor:

31 $$\left\{ \begin{gathered} Y{ = }f\left( \theta \right) \hfill \\ Z{ = }g\left( \theta \right) \hfill \\ \end{gathered} \right.$$

32 $$\left\{ \begin{gathered} \ddot{Y}{ = }\frac{{{d}(\omega \cdot \frac{{{d}Y}}{{{d}\theta }})}}{{{d}t}}{ = }\omega^{2} f^{\prime\prime}\left( \theta \right) \hfill \\ \ddot{Z}{ = }\frac{{{d}(\omega \cdot \frac{{{d}Z}}{{{d}\theta }})}}{{{d}t}}{ = }\omega^{2} g^{\prime\prime}\left( \theta \right) \hfill \\ \end{gathered} \right.$$

where $\theta$ and $\omega$ represent the angular displacement and angular velocity of the DC motor respectively;$\dot{Y}$ and $\dot{Z}$ are the velocity component of the centroid of CGM mechanism;$f^{\prime\prime}\left( \theta \right)$ is the second derivative of $f\left( \theta \right)$ for $\theta$ and the same as $g^{\prime\prime}\left( \theta \right)$.

The ZMP dynamic stability analysis of BGM mechanism is as follows:

33 $$\left\{ \begin{gathered} x_{zmp} = X - \frac{{\ddot{X} \cdot Z}}{{(\ddot{Z} + g)}} \hfill \\ y_{zmp} = Y - \frac{{\ddot{Y} \cdot Z}}{{(\ddot{Z} + g)}} \hfill \\ \end{gathered} \right.$$

where the physical meaning of each symbol is the same as Eq. (30), and $\ddot{X}$ is the acceleration of the centroid of entire mechanism in the $X$ direction; Compared with the CGM mechanism, it can be seen that in order to ensure smooth movement of BGM mechanism, it is necessary to analyze from two dimensions of ZMP, i.e.$x_{zmp}$ and $y_{zmp}$.

As for NCGM mechanism, in Eqs. (22–25), the relationship between overall centroid coordinate and angular displacement of each part can be summarily expressed as follows:

34 $$\left\{ \begin{gathered} Y{ = }\Phi (\theta_{1} ,\theta_{2} ,\theta_{1}^{\prime } ,\theta_{2}^{\prime } ) \hfill \\ Z{ = }\Psi (\theta_{1} ,\theta_{2} ,\theta_{1}^{\prime } ,\theta_{2}^{\prime } ) \hfill \\ \end{gathered} \right.$$

where $\theta_{1}$ and $\theta_{2}$ are the angular displacement of gear relative to the retainer,$\theta_{1}^{\prime }$ and $\theta_{2}^{\prime }$ are the angular displacement of the moving gear relative to horizontal plane; According to transmission characteristics of the second order non-circular gear, i.e. Equations (20)–(21),$\theta_{2}$,$\theta_{1}^{\prime }$ and $\theta_{2}^{\prime }$ can be represented by $\theta_{1}$; Therefore,$\ddot{Y}$ and $\ddot{Z}$ are the acceleration component of centroid, it can also be calculated by Eq. (30).

#### Analyze the influencing factors of ZMP

In order to ensure the stability of mobile mechanism, this paper takes the CGM mechanism as an example, and studies the influence of angular velocity, quality and position of balance piece. In order to facilitate the explanation of motion process of the gear-type mobile mechanism, the contact between driven gear and surface, and the motion process of driving gear rotating around driven gear are recorded as Phase 1; Similarly, the contact between driving gear and ground, and the motion of driven gear rotating around driving gear, is recorded as Phase 2.

**Figure 6:**
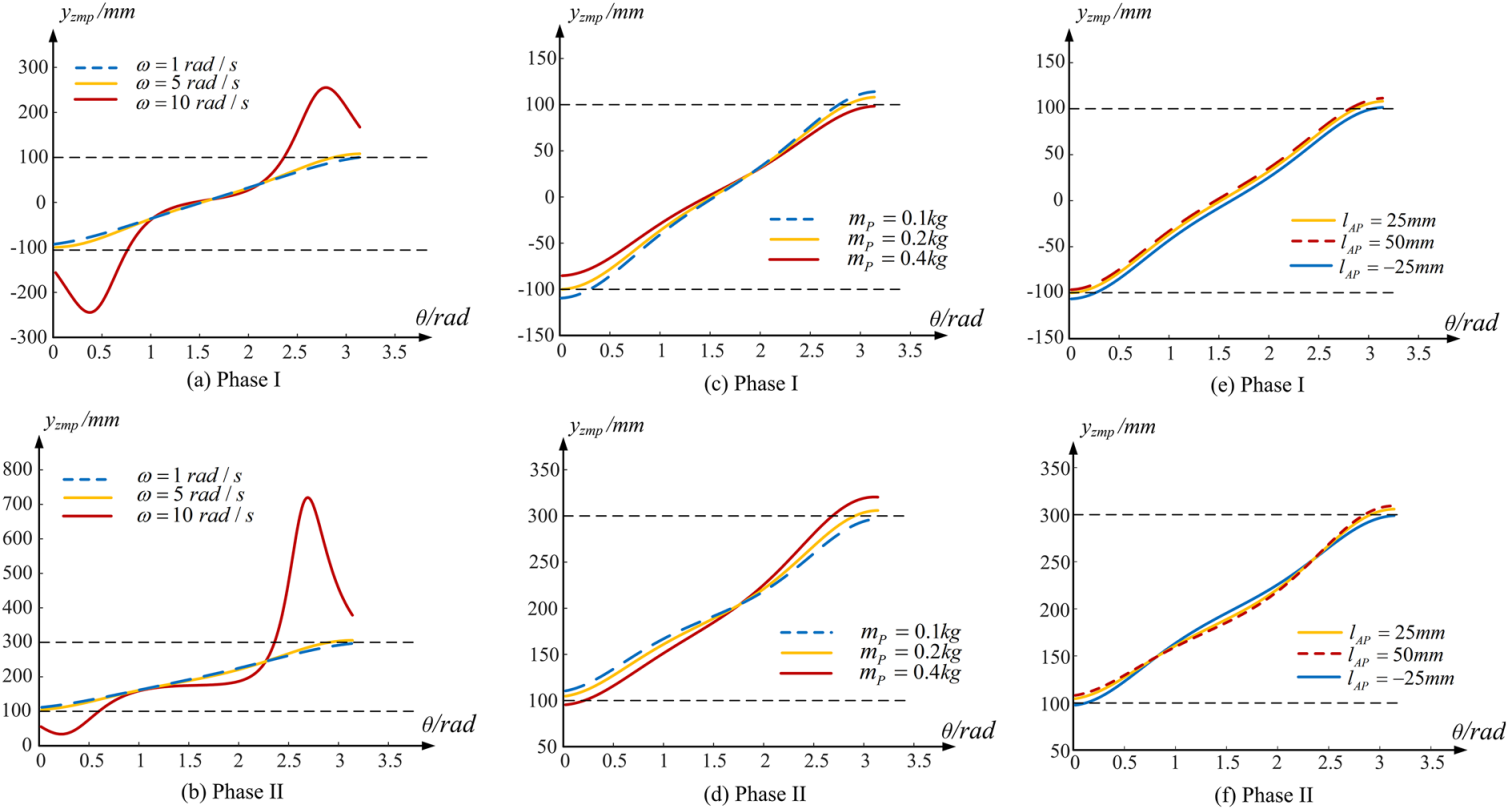
Influencing factors of ZMP trajectory.

The support area of CGM mechanism is divided into two parts, the first half of the support area is $\left( { - 100\;{\text{mm}},100\;{\text{mm}}} \right)$, and the second half of the support area is $\left( {100\;{\text{mm}},300\;{\text{mm}}} \right)$. From Fig. 6a,b, it can be seen that the movement speed has a significant impact on the motion stability of the CGM mechanism. When the angular velocity is $\omega = 1 \, rad/s$, the mechanism can start normally, and there will be no counter-rotating movement between the two gears, and there is almost no impact when landing. With the increase of the angular velocity to five times, it can be found that $\user2{y}_{zmp}$ moves towards the edge of the support area, and its landing height also increases. If the angular velocity continues to increase significantly, the CGM mechanism will not move. However, we can find from Fig. 6c–f that the mass of the balance piece $m_{P}$ and the distance between it and the rotating shaft of gear type mobile mechanism $l_{AP}$ will also change the $y_{{zmp}}$ curve. Therefore, the movement stability of the gear-type mobile mechanism can be guaranteed within a certain range by adjusting the above two parameters.

### Comparison of the gear-type mobile mechanism

**Figure 7:**
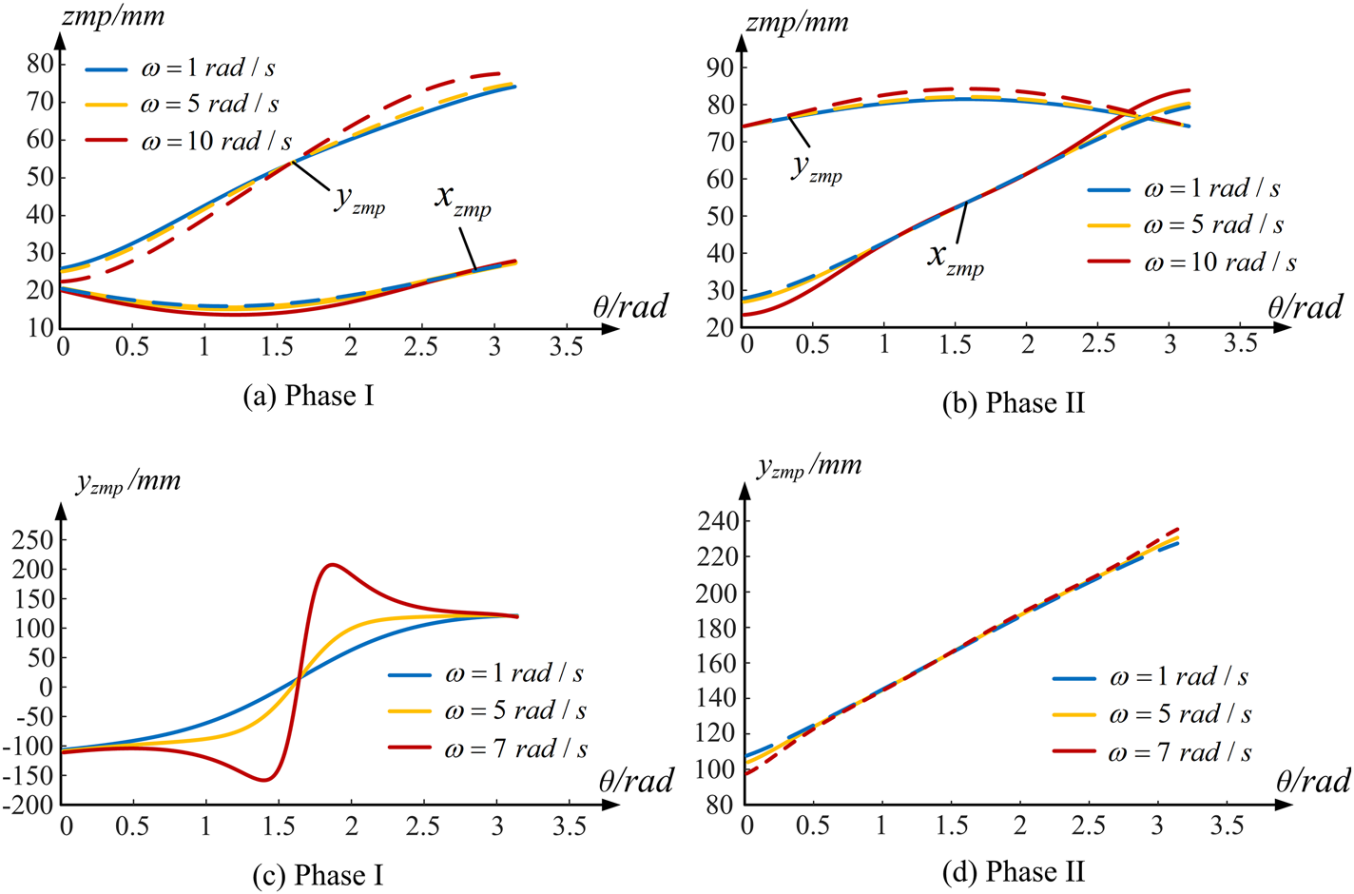
The influence of angular velocity on ZMP trajectory of different geared-type mobile mechanisms.

Figure 7a,b shows ZMP of BGM mechanism, and its support area is within $(0\;{\text{mm}},100\;{\text{mm}})$ and $(0\;{\text{mm}},20\;{\text{mm}})$ respectively. The projection of ZMP space curve on the *xAy* plane has components not only in the x-axis direction, but also in the y-axis direction. The BGM mechanism has a good kinematic characteristics, and it’s motion stability in the the whole cycle is less affected by the motion parameters. The ZMP changes gently and is always in the support area with the angular velocity changes, which will lead to a persistence in stability. Compared with the CGM mechanism, the BGM mechanism has better anti-interference ability when facing the uncertainty of the external environment and the unsteady DC motor speed. Admittedly, there is ZMP offset along the small end of the bevel gear during its movement. However, it can be seen that the offset is not large and it is little affected by its angular velocity, so the influence of offset can be eliminated by adding a base plate.

Figure 7c,d shows the ZMP of NCGM mechanism, and its support area is within $( - 100\;{\text{mm}},100\;{\text{mm}})$ and $(100\;{\text{mm}},250\;{\text{mm}})$ respectively. As same as the above two types of gear type mobile mechanisms, too high motor rotation speed will cause the opposite rotation between gears at the initial stage of motion. If the ZMP is located outside the support area at the starting position, adjusting the mass of the balance piece or moving it to an appropriate place can make the ZMP return to the support area. Simultaneously, it can be seen from the comparison between Figs. 6a and 7a that the angle of ZMP surge caused by excessive angular velocity has changed significantly. The CGM mechanism appears in the initial and final stages, while the NCGM mechanism is closer to the intermediate stage. In other words, different pitch curve shapes correspond to different ZMP slopes, and the stability of gear-type mobile mechanism will change accordingly.

Because the NCGM mechanism designed in this paper installs the DC motor and balance piece on the short shaft non-circular gear, its motion stability in the first half of cycle is very sensitive to the motor’s rotation speed. As shown in Fig. 7d, the concentration of mass leads to the insensitivity of the NCGM mechanism to the speed change during the motion cycle.

#### Prototypes and experiments

**Figure 8:**
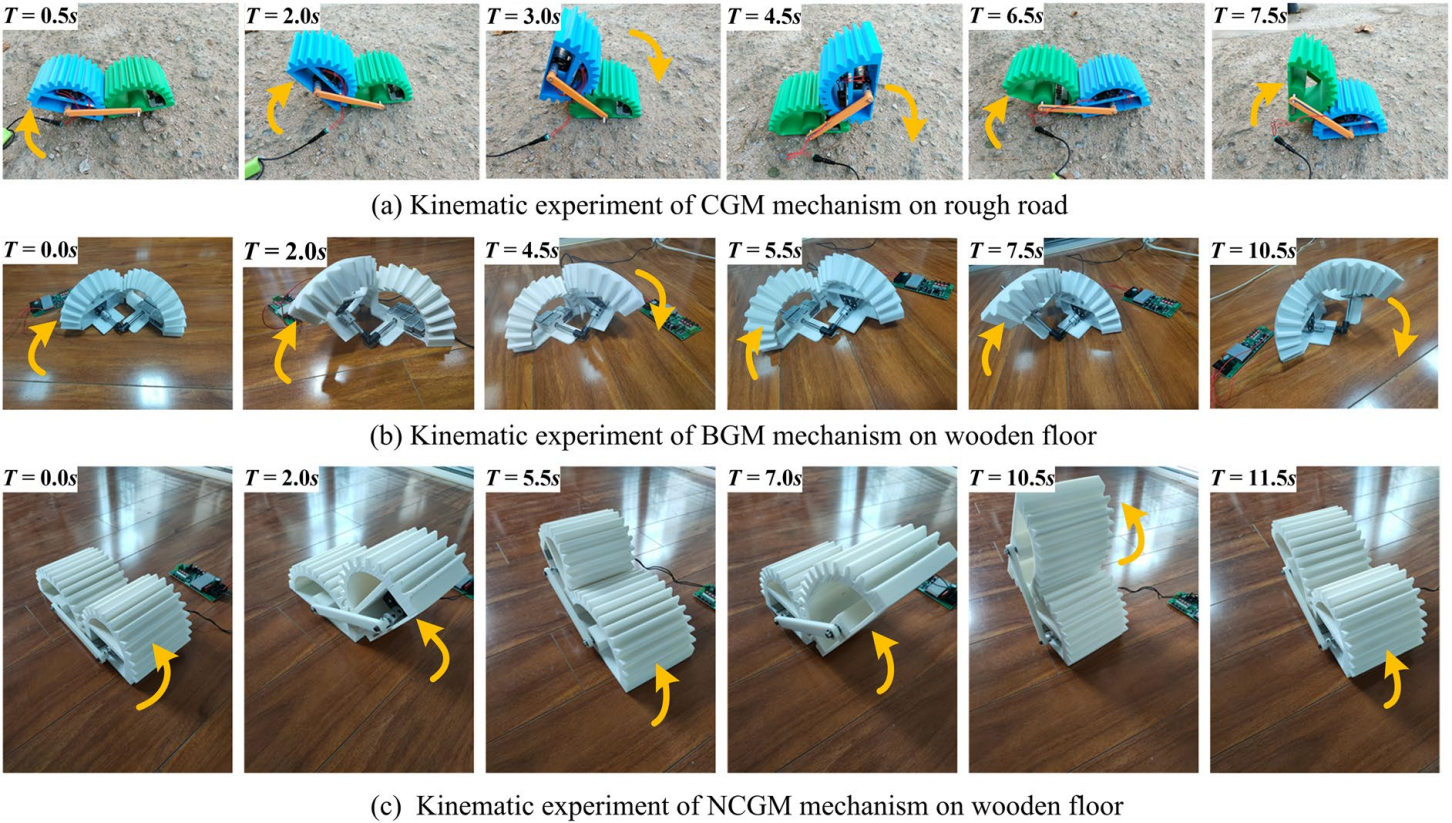
The rough road experiment of CGM mechanism.

In order to verify the mobile performance of gear-type mobile mechanism designed in this paper, three experimental prototypes of the gear-type mobile mechanism were fabricated by 3D printing. Figure 8 shows the motion experiment of the gear-type mobile mechanism. Figure 8a shows the movement of CGM mechanism on rough road surface, Figs. 8b,c show the movement experiment of BGM mechanism and NCGM mechanism on wooden smooth floor respectively. Table 1. provides the design parameters for gear-type mobile mechanism.

**Table 1:** Prototype specification and design parameters.

Robot	Weight (kg)	Motor	Module	Center distance/Installation distance (*mm*)
CGM mechanism	2	DC 12 V; ${5 }r{\text{/min}}$	7	200
BGM mechanism	2	DC 12 V; ${5 }r{\text{/min}}$	8	100
NCGM mechanism	3	DC 12 V; ${5 }r{\text{/min}}$	6	175

#### Potential application scenarios

Many mobile mechanisms have no way to face the plane with large inclination angle. In the above analysis, it can be found that the moving platform rotates around the fixed platform, and at the same time, it is carrying out autobiographical movement as well. And because the gears always keep the meshing relationship, the rotation angle of the moving platform can be controlled by DC motor.

The tooth groove side is the contact surface between the mobile mechanism and the outside, which is generated by a complete gear cut from the middle. Increasing the adsorption with the ground can effectively improve the stability of the mobile mechanism. Of course, it has been proved through analysis and experiments that no other equipment is needed to increase the adsorption force when moving on the horizontal plane. It is necessary to increase the adsorption with the contact surface when moving in a vertical plane or with a maximum inclination angle. The following engineering scenarios demonstrate application examples of the CGM mechanism in over-obstruct.

**Figure 9:**
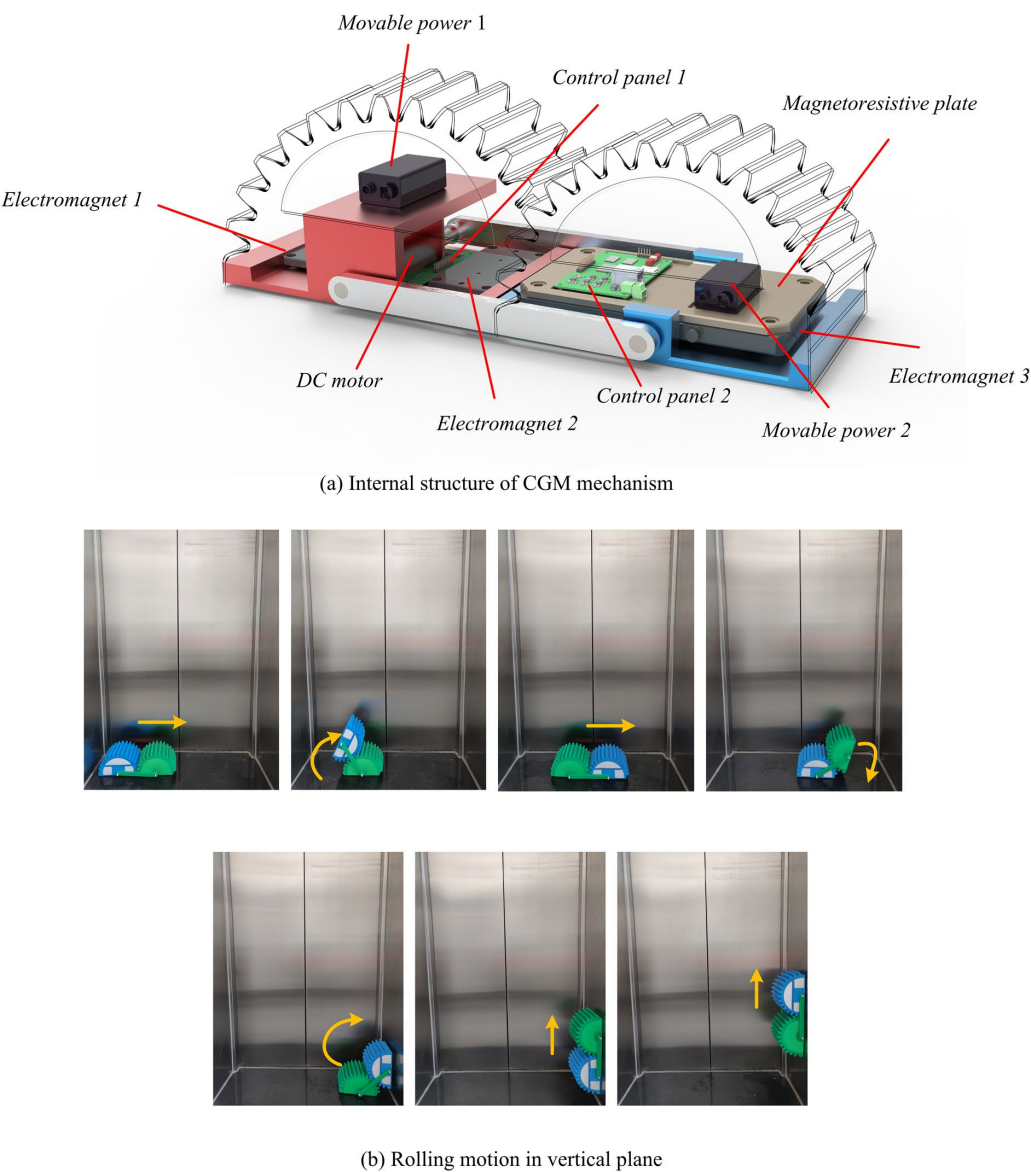
Obstacle-crossing ability experiment.

The internal structure of CGM mechanism applied for moving on the plane with a large inclination angle is shown in Fig. 9a. There are five main components, namely electromagnet, mobile power, control panel, magnetoresistive plate and DC motor. By controlling the forward and reverse rotation of DC motor, moving platform and fixed platform of gear-type mobile mechanism can alternately roll forward. When the electromagnet is powered on, it will generate magnetic force to adsorb on the inclined plane, and when the power is off, it can release the adsorbed platform again. Magnetoresistive plate can reduce the interference of magnetic force on direct contact components.

As shown in Fig. 9b, CGM mechanism moves on the vertical plane through its own rolling and the adsorption of tooth groove surface. The CGM mechanism gradually approaches the right vertical plane from the initial left position. Then is the preparation stage before contacting the vertical plane. At this stage, the CGM mechanism adjusts the tooth surface of the mobile platform to a position parallel to the vertical plane and maintains this state for a period of time. When the mobile platform contacts the vertical plane, it immediately generates an adsorption force to firmly connect the mobile platform with the vertical plane. Next, the DC motor rotates in reverse direction, and the fixed platform in the previous process becomes a mobile platform and rotates around another gear. From then on, the CGM mechanism is completely on the vertical plane and continues to move along the vertical plane through the forward and reverse rotation of the DC motor by 180°.

## Conclusions

In this research, our goal is to design and implement a novel mobile mechanism, which is capable of performing rolling with a single actuator, as well as performing in different ways based on gear structure while rolling. The models of CGM mechanism (linear motion), the BGM mechanism (steering motion) and the NCGM mechanism (variable motion) are established respectively. Based on the screw theory, the DOF of the gear-type mobile mechanism are calculated, and the influence of the centroid trajectory on the motion stability is discussed. Simultaneously, the stability analysis is carried out through the ZMP theory, and the necessary conditions for no opposite rotation are obtained. Then, the effects of various parameters on the moving ability of different gear-type mobile mechanisms are compared and studied as well. Furthermore, the prototype of gear-type mobile mechanism is made, the motion test experiment was carried out on real ground to verify the feasibility of design and the effectiveness of analysis. It has the advantages of simple mechanical structure and reliable movement.

## Data Availability

The datasets generated and/or analysed during the current study are not publicly available due [REASON WHY DATA ARE NOT PUBLIC] but are available from the corresponding author on reasonable request.
